# Personalizing cholesterol treatment recommendations for primary cardiovascular disease prevention

**DOI:** 10.1038/s41598-021-03796-6

**Published:** 2022-01-07

**Authors:** Ashish Sarraju, Andrew Ward, Jiang Li, Areli Valencia, Latha Palaniappan, David Scheinker, Fatima Rodriguez

**Affiliations:** 1 Division of Cardiovascular Medicine and the Cardiovascular Institute, Center for Academic Medicine, Stanford University School of Medicine, 453 Quarry Road, Stanford, CA USA; 2 Department of Electrical Engineering, Stanford University, Stanford, CA USA; 3 Palo Alto Foundation Research Institute, Palo Alto, CA USA; 4 Division of Primary Care and Population Health, Stanford University School of Medicine, Stanford, CA USA; 5 Department of Management Science and Engineering, Stanford University School of Engineering, Stanford, CA USA; 6 Division of Pediatric Endocrinology, Stanford University School of Medicine, Stanford, CA USA

**Keywords:** Cardiology, Risk factors, Machine learning, Disease prevention

## Abstract

Statin therapy is the cornerstone of preventing atherosclerotic cardiovascular disease (ASCVD), primarily by reducing low density lipoprotein cholesterol (LDL-C) levels. Optimal statin therapy decisions rely on shared decision making and may be uncertain for a given patient. In areas of clinical uncertainty, personalized approaches based on real-world data may help inform treatment decisions. We sought to develop a personalized statin recommendation approach for primary ASCVD prevention based on historical real-world outcomes in similar patients. Our retrospective cohort included adults from a large Northern California electronic health record (EHR) aged 40–79 years with no prior cardiovascular disease or statin use. The cohort was split into training and test sets. Weighted-K-nearest-neighbor (wKNN) regression models were used to identify historical EHR patients similar to a candidate patient. We modeled four statin decisions for each patient: none, low-intensity, moderate-intensity, and high-intensity. For each candidate patient, the algorithm recommended the statin decision that was associated with the greatest percentage reduction in LDL-C after 1 year in similar patients. The overall cohort consisted of 50,576 patients (age 54.6 ± 9.8 years) with 55% female, 48% non-Hispanic White, 32% Asian, and 7.4% Hispanic patients. Among 8383 test-set patients, 52%, 44%, and 4% were recommended high-, moderate-, and low-intensity statins, respectively, for a maximum predicted average 1-yr LDL-C reduction of 16.9%, 20.4%, and 14.9%, in each group, respectively. Overall, using aggregate EHR data, a personalized statin recommendation approach identified the statin intensity associated with the greatest LDL-C reduction in historical patients similar to a candidate patient. Recommendations included low- or moderate-intensity statins for maximum LDL-C lowering in nearly half the test set, which is discordant with their expected guideline-based efficacy. A data-driven personalized statin recommendation approach may inform shared decision making in areas of uncertainty, and highlight unexpected efficacy-effectiveness gaps.

## Introduction

Atherosclerotic cardiovascular disease (ASCVD) remains the leading cause of death in the United States^1^. Cholesterol management through statin therapy is the cornerstone of ASCVD prevention^2^. The primary goal of statin therapy is to adequately and sustainably decrease low density lipoprotein (LDL-C) levels and ASCVD risk^3^. For primary prevention, the 2018 American College of Cardiology (ACC)/American Heart Association (AHA) guidelines recommend estimating 10-year ASCVD risk using the Pooled Cohort Equations (PCE), a guideline-endorsed risk calculator, to guide statin therapy decisions.

High-intensity statins decrease LDL-C up to 50% in ideal, monitored clinical trial settings^4^. Thus, for patients with the highest ASCVD risk, guidelines recommend high-intensity statin therapy^2^. However, recommendations are less definitive for other groups such as patients with borderline or intermediate ASCVD risk, for whom decisions depend on individualized risk–benefit assessment. Recommendations may also be less certain in diverse racial/ethnic groups including heterogenous Hispanic or Asian populations. For example, certain Asian groups may be at higher risk for statin-related side effects when prescribed high-intensity statins such as rosuvastatin^2^. In real-world settings outside of randomized trials, statin effectiveness for LDL-C lowering can be limited by medication nonadherence, statin-related symptoms, patient- or provider-concerns, and nocebo effects^2,5–10^. Thus, prescribing practices and LDL-C lowering from statin by intensity differ from contemporary practice guidelines.

For a given patient, determining the statin recommendation associated with optimal real-world outcomes—and overcoming discrepancies between guidelines and real-world effectiveness—is crucial for decision-making. To bridge this gap, aggregate historical real-world data may help understand prior treatment responses and guide personalized decision making by incorporating real-world outcomes in areas of therapeutic uncertainty^11^. In particular, machine learning (ML) can leverage aggregate outcomes to understand prior responses to therapies, which may help guide patient-clinician discussions^11–16^. These approaches are likely most helpful in primary ASCVD prevention, when there may be more uncertainty and ambiguity about the best treatment options, particularly in understudied racial/ethnic groups. The objective of this study was to develop an EHR-based ML approach to examine historical outcomes of LDL-C reduction under different lines of statin therapy and recommending the statin therapy associated with the greatest relative cholesterol reduction for similar patients. We hypothesized that there would be differences in personalized statin recommendations across patients which are not fully explained by the expected LDL-C lowering of each statin intensity.

## Methods

### Cohort

All research was performed in accordance with relevant guidelines and regulations. The study was approved by the Stanford University and Sutter Health System Institutional Review Board, who determined that the research does not involve human subjects and granted a waiver of consent based on the nature of the project, including the use of previously collected, de-identified data. Patients from a Northern California-based health system (Sutter Health) were included if they had with at least 2 outpatient visits at least 1 year apart between 2009 and 2018. Inclusion criteria included patients aged 40 through 79 years with at least 1 follow-up LDL-C value, no prior cardiovascular disease (CVD), not on statins at baseline, on stable statin intensities during follow-up, and available data for total cholesterol, high density lipoprotein cholesterol (HDL-C), and blood pressure measurements. If there were no cholesterol laboratory results before a patient’s index date, the index date was shifted to the date of the first cholesterol lab result. Prior CVD was defined by the International Classification of Diseases, 9th and 10th revision (ICD-9-CM/ICD-10-CM) coding scheme as per the 2013 American College of Cardiology/American Heart Association Guideline on the Assessment of Cardiovascular Risk (CV conditions excluded from the derivation cohort of the PCE and from PCE application; Supplementary Table 1)^17^.

### Patient variables

All variables that are used for PCE estimates were extracted from the EHR and included: age, race/ethnicity, total cholesterol, high-density lipoprotein cholesterol (HDL-C), smoking history, systolic blood pressure, treatment for high blood pressure (antihypertensive medications), and diabetes^17^. Missing race was inferred based on the Social Security Record database^18^. Diabetes status was identified by either a diagnosis of diabetes (ICD-9-CM: 250.*; ICD-10-CM: E11*, Z79.4, Z79.84) or a diabetes medication (GPI2: 27) prescribed on or prior to index date. For total cholesterol, HDL-C, systolic blood pressure, and smoking status, the most recent value on or before the index date was used. The most recent height, weight, and diastolic blood pressure measurements before each patient’s index date were also included. Antihypertensive medications were determined on the date of the patient’s blood pressure measurement (GPI codes beginning with 33, 34, 36, 37, 4013, or 4016). Statin intensity was defined as low, moderate, or high based on the agent and dose, according to ACC/AHA guidelines based on expected LDL-C lowering (Supplementary Table 2)^17^.

Additional variables were selected based on EHR availability and relevance to CVD risk (Supplementary Table 3). Variables were extracted one year prior to the index date. Medical conditions were extracted from the EHR problem list coded in ICD-9-CM/ICD-10-CM and grouped into 283 categories using the Clinical Classification Software (CCS)^19^. Self-reported family (parents or sibling) medical histories were extracted from the family history section of the EHR. CCS and family history conditions were coded as binary variables. Medication prescription information was obtained by using prescriptions’ GPI codes (GPI4). The total number of medication prescriptions in the prior year was included as a variable. The total number of laboratory tests ordered and the total number which returned “abnormal” results were included. Socioeconomic variables were derived from patient addresses and included census block group level indicators of educational attainment and median household income. To account for differences in healthcare utilization, the number of primary care, urgent care, specialty, and other (e.g., ancillary) service care visits in the previous year were captured.

### Outcome

The outcome was the relative percentage (%) reduction in LDL-C level at 1-year follow-up across different statin therapy decisions (no statins, low-intensity, moderate-intensity, high-intensity). The recommended statin prescription was defined as the intensity resulting in the highest percentage-decrease in LDL-C level at 1 year.

### Model development

The patient cohort was split into training (index date before 2015) and held-out test (index date in 2015 or later) sets. Training and test sets were defined by index date to reflect typical real-world practice wherein algorithms are first developed with existing older patient data and applied to more contemporary patients. We first developed weighted-K-nearest neighbor (wKNN) regression models in the training set. For each statin therapy decision, a separate weighted Lasso regression model was trained using EHR variables of all patients who received the specified line of statin therapy on the outcome of relative (%) 1-year LDL-C reduction^20^. The magnitude of the resulting regression model coefficients for each therapy decision were used as weights to define treatment-specific weighted Euclidean distances between any two candidate patients.

To select K, fivefold cross validation was used among the training patients. For each line of statin therapy, Lasso models were trained on non-held-out patients, and patients in the held-out fold with that line of therapy were matched to K patients in the non-held-out fold for differing values of K. For each patient in the held-out fold, the averaged 1-year relative (%) LDL-C reduction of the K-nearest patients was used to estimate the LDL-C reduction of the held-out fold patient, and then compared to the true LDL-C reduction for that patient. The values of K which minimized the mean-squared error between the estimated and true 1-year relative (%) LDL-C reduction across all 5 held-out folds were used in each of the four final wKNN models (one for each statin therapy). After all values of K were selected, the Lasso models were retrained on all training set patients to determine final model weights.

In the test set, for each patient, the trained wKNN model for the corresponding statin therapy was used to identify the K-nearest similar patients in the training set. Relative (%) LDL-C reduction at 1 year for each line of statin therapy was estimated by averaging the 1-year relative (%) LDL-C reduction for K-nearest training-set patients. The statin therapy associated with the highest estimated 1-year relative (%) LDL-C reduction in similar patients was recommended. Each statin intensity recommendation group was profiled with respect to baseline characteristics. Analyses were performed in Python 3.7 using the scikit-learn package, version 0.21.2^21^.

## Results

**Figure 1:**
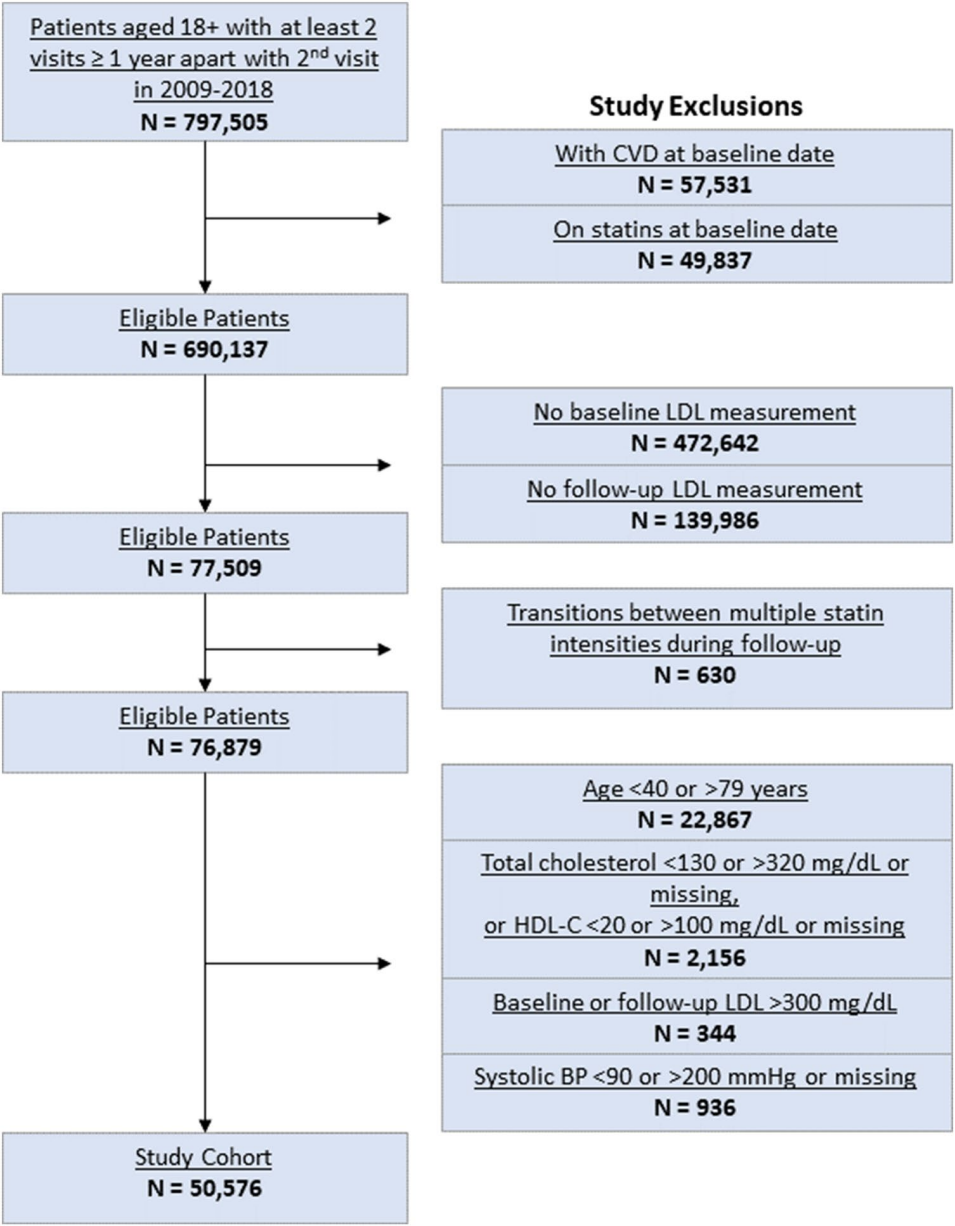
CONSORT cohort selection diagram. *BP* Blood pressure, *CVD* Cardiovascular disease, *HDL-C* High density lipoprotein cholesterol, *LDL-C* Low density lipoprotein cholesterol.

The final study cohort consisted of 50,576 patients, split into 42,193 patients in the training set and 8383 patients in the test set (Fig. 1). Patients had an overall mean age [± standard deviation (SD)] of 54.6 ± 9.9 years and 55% were female. The cohort was enriched with understudied racial/ethnic groups, including 32% Asian and 7.4% Hispanic patients (Table 1). Average baseline LDL-C and total cholesterol (± SD) were 121.3 ± 30 mg per deciliter (mg/dL) and 200.7 ± 34 mg/dL, respectively. A minority of patients were on antihypertensive medications (28%), had type 2 diabetes (10%), or were current smokers (4.2%). The average 5-year PCE-derived ASCVD risk was 3.0% ± 4.0%, and the average follow-up 1-year LDL-C was 121 mg/dl in the overall cohort. Most patients (92%) did not receive statin therapy. Approximately 2.6% (1,330), 5% (2,519), and 0.7% (352) of patients received low-intensity, moderate-intensity, and high-intensity statins, respectively.

**Table 1:** Baseline characteristics of the cohort.

	Training data*	Test data^†^	Total
N	42,193	8383	50,576
Age	54.63 ± 9.79	54.40 ± 10.12	54.59 ± 9.85
Female	22,983 (54)	4593 (55)	27,576 (55)
Baseline LDL-C	121.82 ± 29.90	118.88 ± 31.80	121.33 ± 30.25
Total cholesterol	200.47 ± 33.70	201.75 ± 35.34	200.68 ± 33.98
HDL cholesterol	54.26 ± 14.69	57.89 ± 15.82	54.86 ± 14.95
Systolic BP	124.07 ± 16.48	124.10 ± 17.38	124.08 ± 16.63
Diastolic BP	77.00 ± 10.32	76.49 ± 10.44	76.91 ± 10.34
History of type 2 diabetes	4205 (10)	1025 (12)	5230 (10)
Antihypertensive medications	11,759 (28)	2266 (27)	14,025 (28)
Current smoker	1739 (4.1)	373 (4.4)	2112 (4.2)
5-year ASCVD risk estimate (%)	3.0 ± 4.0	3.0 ± 4.0	3.0 ± 4.0
**Race**			
African American	753 (1.8)	131 (1.6)	884 (1.7)
Asian	13,322 (32)	2938 (35)	16,260 (32)
Hispanic	3098 (7.3)	663 (7.9)	3761 (7.4)
Non-Hispanic White	20,868 (49)	3163 (38)	24,031 (48)
Other	706 (1.7)	261 (3.1)	967 (1.9)
Unknown	3446 (8.2)	1227 (15)	4673 (9.2)
**Statin at follow-up**			
Low-intensity Statin	1250 (3)	80 (0.95)	1330 (2.6)
Moderate-intensity Statin	1963 (4.7)	556 (6.6)	2519 (5)
High-intensity Statin	241 (0.57)	111 (1.3)	352 (0.7)
No Statin	38,739 (92)	7636 (91)	46,375 (92)

*ASCVD* Atherosclerotic cardiovascular disease, *BP* Blood pressure, *HDL* High density lipoprotein, *LDL-C* Low-density lipoprotein cholesterol.

*Index date before 2015.

^†^Index date 2015 or later.

**Figure 2:**
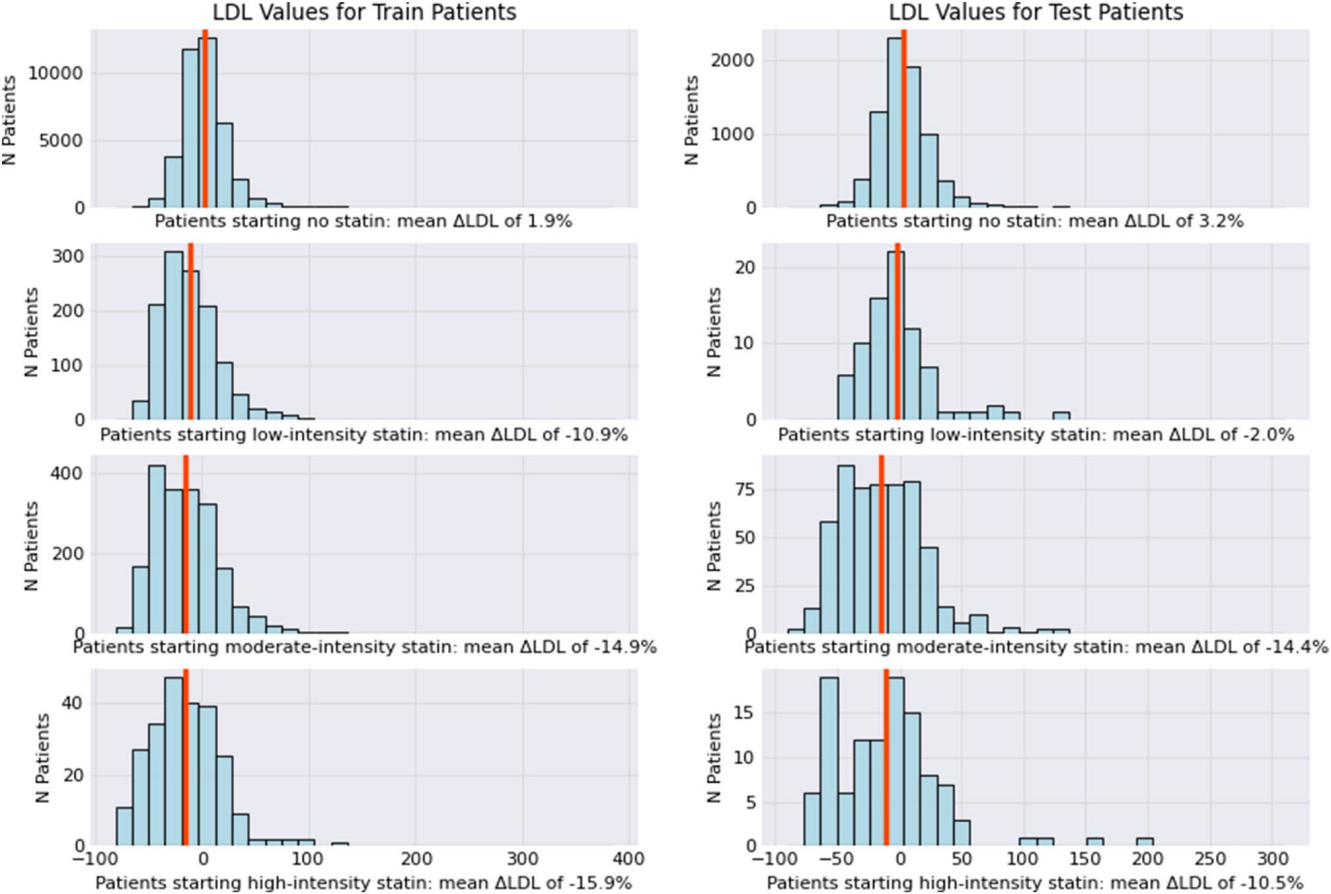
Low density lipoprotein cholesterol responses at 1-year across different statin therapies in the training and test cohorts. *LDL-C* Low density lipoprotein cholesterol; *ΔLDL-C* Change in LDL-C (%).

A total of 57 EHR variables were used for the wKNN regression models. Across the 42,193 training data set patients, high-intensity statin prescriptions were associated with the greatest average relative (%) reduction in LDL-C (15.9%), compared with moderate-intensity (14.9%), or low-intensity therapy (10.9%) (Fig. 2). Among the 8383 test set patients, at baseline, moderate-intensity statins were associated with the highest relative (%) LDL-C reduction (14.4%), compared with high- (10.4%) or low-intensity (2%) at baseline (Fig. 2).

**Figure 3:**
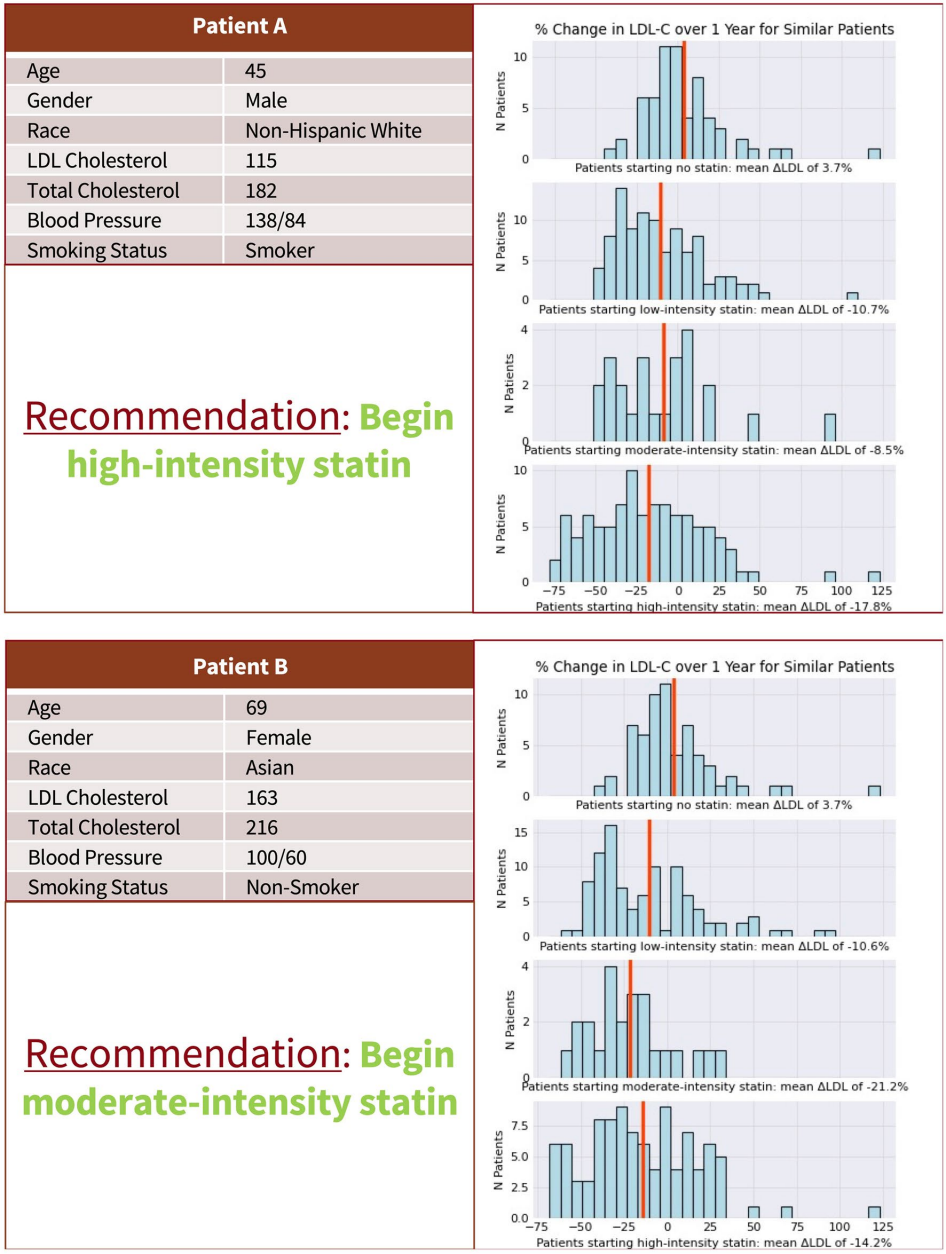
Examples of personalized statin recommendations. Sample recommendations are visualized for patients who were recommended high-intensity therapy (**A**) and moderate-intensity therapy (**B**) based on optimal relative LDL-C lowering across different lines of statin therapy in similar patients, determined through weighted-K-nearest-neighbor regression models. Selected, common baseline patient characteristics are described alongside each recommendation for identification purposes. *LDL-C* Low density lipoprotein, *ΔLDL-C* Change in LDL-C.

Overall, in test patients, 52%, 44%, and 4% of patients were recommended to begin high-intensity, moderate-intensity, and low-intensity statins, respectively, based on personalized relative (%) LDL-C reduction outcomes. No patients received a recommendation for no statin therapy. With these recommendations, the LDL-C values at 1-year follow-up in similar patients were 18.4% ± 22.9% lower per patient, on average, with an average reduction of 14.9% in those recommended low-intensity therapy, 20.4% in those recommended moderate-intensity, and 16.9% in those recommended high-intensity therapy (Table 2). For each patient, the personalized recommendation approach described and visualized the anticipated % LDL-C lowering across all lines of statin therapy (Fig. 3) and generated a therapy recommendation. Characteristics of each statin intensity recommendation group across the EHR, including age, gender, race/ethnicity, ASCVD risk factors, and socioeconomic and healthcare utilization variables, are outlined in Table 2.

**Table 2:** Characteristics of test set patients according to recommended statin therapy.

Characteristic [At baseline unless otherwise specified; N (%) unless otherwise specified]	Low-intensity statin recommended	Moderate-intensity statin recommended	High-intensity statin recommended
Total N	399	3719	4325
Age (Mean ± SD), years	54.3 ± 10	54.3 ± 10.1	54.5 ± 10.1
Female	122 (36)	2251 (61)	2220 (51)
Non-Hispanic White	112 (33)	1468 (39)	1545 (36)
Black	8 (2.4)	56 (1.5)	67 (1.5)
Hispanic	24 (7.1)	299 (8)	340 (7.9)
Asian	112 (33)	1274 (34)	1552 (36)
LDL-C (Mean ± SD), mg/dl	110.5 ± 28.7	119.6 ± 31.2	118.9 ± 32.5
Average predicted LDL-C reduction at 1 year (%)	14.9	20.4	16.9
Systolic Blood pressure (mean ± SD), mmHg	123.9 ± 16.3	123.7 ± 17.4	124.5 ± 17.5
History of Type 2 Diabetes	49 (14)	435 (12)	541 (13)
On antihypertensive medication	81 (24)	988 (27)	1197 (28)
Current smoking	27 (8)	155 (4.2)	191 (4.4)
PCE-derived 5-year ASCVD risk estimate (%)	3 ± 4	3 ± 4	3 ± 4
Total medications prescribed in prior year	2.0 ± 2.0	2.3 ± 2.5	2.3 ± 2.5
N primary care visits in prior year	1.4 ± 1.7	1.4 ± 1.7	1.4 ± 1.5
Median Household Income (Mean ± SD), US Dollars	95,987 ± 50,331	93,107 ± 50,478	94,094 ± 50,339
Percent with up to a bachelor’s degree, %	67 ± 28	68 ± 28	68 ± 28

*ASCVD* Atherosclerotic cardiovascular disease, *LDL-C* Low-density lipoprotein cholesterol, *PCE* Pooled cohort equations, *SD* Standard deviation, *US* United States.

## Discussion

Using aggregate patient data from a cohort of similar patients, we developed a personalized statin recommendation approach for primary ASCVD prevention that estimated LDL-C outcomes across different statin treatment strategies and recommended the intensity associated with the highest LDL-C reduction. The approach identified patients who were recommended moderate- or low-intensity statins—rather than high-intensity statins—based on LDL-C lowering outcomes in similar patients, which is discordant with their expected guideline-based efficacy. These findings support the importance of using real-world data to identify patients at risk for suboptimal LDL-C lowering and to inform shared decision-making in areas of clinical uncertainty.

Guideline recommendations largely rely on efficacy outcomes from randomized clinical trials. However, discrepancies between trial efficacy and real-world statin effectiveness represent an alarming gap in primary prevention, with multifactorial causes including patient-level, clinician-level and system-level factors^5–7,22^. In the Lipid Treatment Assessment Project, only 38% of 4888 patients with hyperlipidemia met prespecified targets of LDL-C under 100 mg/dL after 3 months of lipid therapy^23^. The International Cholesterol Management Practice Study (ICLPS) of patients at cardiovascular risk found that 32.1% of very high-risk, 51.9% of high-risk, and 55.7% of moderate-risk patients achieved their LDL-C lowering goals on treatment^24^. In the centralized pan-European survey on the under-treatment of hypercholesterolemia (CEPHEUS) study, only 55.3% achieved their LDL-C goal, and in the Dyslipidemia International Study (DYSIS), approximately 27% of patients achieved their LDL-C goal^8,9^. Our findings extend this literature demonstrating real-world statin response gaps in primary CVD prevention. Across 42,193 patients in our training dataset, high-intensity statin use was associated with only a 15.9% average decrease in LDL-C levels at 1 year. By contrast, in the Pravastatin or Atorvastatin Evaluation and Infection Therapy–Thrombolysis in Myocardial Infarction 22 (PROVE IT TIMI 22) randomized trial, median LDL-C lowering was 51% with atorvastatin (a high-intensity statin)^4^. Real-world gaps in statin efficacy may reflect differences in adherence and unmeasured confounders in statin intensity and patient selection. Accordingly, our work describes a personalized approach to inform shared decision-making based on real-world outcomes of statin therapy.

Our approach recommended high-intensity statins for over half the test set, which is consistent with their known superior LDL-C lowering efficacy in clinical trials. The approach also identified patients who were recommended low- or moderate-intensity therapy, rather than high-intensity therapy, which is discordant with their expected efficacy based on trials and guidelines. These findings may inform clinical decision-making by signifying possible barriers to higher intensity statins in these groups, including patient factors (such as nonadherence or intolerance with higher statin intensities leading to suboptimal LDL-C lowering) or provider biases (including preference for lower intensity statins). Identifying groups with such guideline-discordant responses is a crucial step to detect and address such patient or provider barriers to optimal LDL-C lowering.

The human component of patient-clinician discussions is the cornerstone of medical decision-making where patients and clinicians discuss benefits, risks, and barriers related to statin use. ML approaches cannot (and should not) replace human shared decision-making, but rather, may help optimize shared decision-making by personalizing these discussions to the individual patient. For example, if our ML approach recommends a low-intensity statin, clinicians and patient may wish to explore the potential reasons why a high-intensity statin was not recommended, such as risk for side effects, risk for nonadherence, or historical provider bias. Such patients may also be “flagged” for close follow-up to ensure long-term statin adherence and persistence^25^. Clinicians may dedicate targeted efforts and conversations to reinforce the importance of statin therapy and strengthen the therapeutic patient-clinician relationship. If statin hesitancy or intolerance is suspected, patients may be considered early for alternative statin dosing strategies such as alternate-day dosing or lower starting doses^25,26^. The use of aggregate real-world data may thus help tailor clinician-patient discussions to personalize shared-decision making efforts in areas of treatment uncertainty. This has been previously studied as a “green button” approach that helped inform patient care when implemented as a pilot, on-demand service at the Stanford Medicine health system^11,27^. Future work should continue to assess such approaches prospectively in clinical practice through EHR integration.

Our approach profiled the three (low-, moderate- and high-intensity) statin recommendation groups across EHR factors including age, gender, race/ethnicity, ASCVD risk factors, socioeconomic status, and healthcare utilization, as reported in Table 2. Intriguingly, these data did not suggest clinically meaningful differences in age, baseline LDL-C, history of type 2 diabetes, estimated ASCVD risk, or Asian race between the three recommendation groups –despite being factors that are relevant to statin intensity decisions based on guidelines. These results raise the hypothesis that these key variables did not drive statin decisions in our cohort. There was a lower proportion of female patients in the low-intensity recommendation group compared with the moderate-intensity or high-intensity recommendation groups (36% versus 60% or 51%, respectively), and a slightly higher proportion of Black patients in the low-intensity recommendation group compared with the moderate or high-intensity recommendation groups (2.4% versus 1.5% or 1.5%, respectively). Personalized data-driven approaches may promote investigation into factors associated with historical real-world responses, which may be of interest to clinicians.

Limitations of our study include a primarily insured Northern-California based health system with patients who may not be generalizable across the United States, and who may be at lower ASCVD risk than under-insured populations—but they may consequently experience more clinical uncertainty regarding statin decisions^28^. Our cohort is also enriched for traditionally underrepresented populations including Asian and Hispanic groups and elderly patients. Race/ethnicity was self-reported or inferred from validated methods, which may contribute to misclassification^18^. Lack of disaggregated data regarding Asian and Hispanic groups may mask important within-group heterogeneity. Though our cohort was large, only 8% were on statin therapy at baseline, reflecting a lower risk population who experience more treatment uncertainty. More patients on statin therapy would have provided larger training datasets for ML models. Recommendations based on real-world data may be affected by unmeasured confounding. Thus, they should be largely viewed as associations between therapy and outcomes rather than evidence of causality. Specifically, real-world data could reflect historical biases and overrepresent traditionally favored therapies. To address this, we visualized outcomes across all lines of therapy to allow users to examine the data underlying recommendations. We also focused on relative LDL-C reduction as a primary endpoint to minimize confounding (rather than absolute LDL-C values, which may affect initial statin decisions) and as a clinically relevant endpoint with a known relationship with ASCVD risk^29^. Our cohort was not restricted to specific primary prevention groups (such as high risk patients) in order to minimize confounding by indication in training data, to include patients with clinical equipoise regarding statin therapy (such as borderline- or low-risk patients), and to increase the study sample size.

In conclusion, in a large multiethnic health system, we developed a personalized statin decision-making approach for primary prevention using real-world data by modeling the statin therapy associated with highest LDL-C reduction in historical patients similar to a candidate patient. In nearly half the test cohort, low- or moderate-intensity statins (rather than high-intensity) were associated with the greatest LDL-C reduction in similar patients, which is discordant with clinical trial and guideline-based efficacy. A data-driven personalized statin decision-making approach may help identify patients at risk for suboptimal statin utilization or responses, may inform shared decision making in the presence of clinical uncertainty, and may provide a pathway to study statin efficacy-effectiveness gaps.

## Supplementary Information

Supplementary Information.

## Data Availability

The datasets generated during and/or analyzed during the current study are available from the corresponding author on reasonable request.
