# Supplementary material for: Personalizing cholesterol treatment recommendations for primary cardiovascular disease prevention
Source: Sci Rep. 2022 Jan 7;12:23. doi: 10.1038/s41598-021-03796-6 (PMC8742083; doi:10.1038/s41598-021-03796-6)
Supplement: Supplementary file 1 — Supplementary Information. [file 41598_2021_3796_MOESM1_ESM.pdf]

Personalizing Cholesterol Treatment Recommendations for Primary Cardiovascular Disease Prevention

Ashish Sarraju[¶], Andrew Ward[¶], Jiang Li, Areli Valencia, Latha Palaniappan, David Scheinker, Fatima Rodriguez[*]

[¶]These authors contributed equally to this work.

*Corresponding author.

Supplementary Information

Supplemental Table 1. Definitions of Cardiovascular Disease.

	ICD-9-CM	ICD-10-CM
Myocardial infarction	410.*	I21.*, I22.*, I23.3, I24.0, I24.9, I25.9, I51.3,
Coronary artery disease	411.*, 413.*, 414.*	I20.*, I23.7, I24.*, I25.*, T82.85,
Ischemic stroke	433.01, 433.11, 433.21, 433.31, 434.81, 433.91, 434.11, 434.91, 436.*	G46.*, I63.*, I67.85, I69.30, I77.89, P91.0, Z86.73
Hemorrhagic stroke	430.*, 431.*, 432.*	I60.*, I61.*, I62.*, I63.89, I67.1, P52.*, P54.8, S06.2*, S06.4*, S06.5*
Atrial fibrillation, heart failure	427.31, 428.*	I27.29, I48.0, I48.1, I48.2, I48.91, I50.*, I51.9, Z86.79
Other cardiovascular disease	412.*, 346.6*, 433.*, 434.*, 435.*, 437.*, 438.*, 440.*, V45.81, or V45.82	G43.6*, G45.*, G46.*, G81.94, G83.9, G93.89, G93.9, G95.19, I25.1, I25.2, I63.*, I65.*, I66.*, I67.* , I68.*, I69.*, I70.*, I72.5, I76, I77.75, I77.89, I77.9, I99.8, M47.01, P91.0, Z98.61

Abbreviations: ICD-9-CM–International classification of diseases, 9th revision; ICD-10-CM–International classification of diseases, 10th revision

Supplemental Table 2. Classification of Statin Intensity.

Cholesterol Lowering Strategies	Definition
Low-intensity statin	Fluvastatin 20-40mg<br>Lovastatin 20mg<br>Simvastatin 10mg<br>Pitavastatin 1mg
Moderate-intensity statin	Atorvastatin 10-20mg<br>Fluvastatin 40mg BID or 80mg XL<br>Lovastatin 40mg<br>Pitavastatin 2-4mg<br>Pravastatin 40-80mg<br>Rosuvastatin 5-10mg<br>Simvastatin 20-40mg
High-intensity statin	Atorvastatin 40-80mg<br>Rosuvastatin 20-40mg

Abbreviations: mg, milligrams

Supplemental Table 3. List of patient features used in the weighted k-nearest neighbor (wkNN) regression model for cross-validation.

Category	Variable
PCE	Age<br>Female<br>HDL cholesterol<br>Total cholesterol<br>History of Type 2 Diabetes<br>Current smoking status<br>On antihypertensive medications<br>Systolic BP<br>Race: African American (Self-reported)<br>Race: Non-Hispanic White (Self-reported)
Clinical Variables	Diastolic BP<br>Height<br>Weight<br>Race: Asian (Self-reported)<br>Race: Hispanic (Self-reported)
Socioeconomic Variables	Median household income<br>Percent with up to a 9th grade education<br>Percent with up to 12th grade education<br>Percent with up to a high school graduation<br>Percent with up to some college<br>Percent with up to an associate's degree<br>Percent with up to a bachelor's degree
Utilization Variables	Number of other service visits<br>Number of primary care visits<br>Number of specialty care visits
Diagnostic Variables	CCS: Alcohol-related disorders<br>CCS: Anxiety disorders<br>CCS: Aortic; peripheral; and visceral artery aneurysms<br>CCS: Cancer of head and neck<br>CCS: Cancer of kidney and renal pelvis<br>CCS: Chronic kidney disease<br>CCS: Chronic ulcer of skin<br>CCS: Deficiency and other anemia<br>CCS: Delirium, dementia, and amnestic and other cognitive disorders<br>CCS: Diabetes mellitus with complications<br>CCS: Diabetes mellitus without complication<br>CCS: Diseases of white blood cells<br>CCS: Epilepsy; convulsions

	CCS: Essential hypertension<br>CCS: Heart valve disorders<br>CCS: HIV infection<br>CCS: Hypertension with complications and secondary hypertension<br>CCS: Nervous approach congenital anomalies<br>CCS: Nonspecific chest pain<br>CCS: Other and ill-defined heart disease<br>CCS: Other connective tissue disease<br>CCS: Other endocrine disorders<br>CCS: Other hematologic conditions<br>CCS: Other infections; including parasitic<br>CCS: Other injuries and conditions due to external causes<br>CCS: Other nervous approach disorders<br>CCS: Other non-traumatic joint disorders<br>CCS: Pancreatic disorders (not diabetes)<br>CCS: Parkinson's disease<br>CCS: Peri-; endo-; and myocarditis; cardiomyopathy (except that caused by tuberculosis or sexually transmitted disease)<br>CCS: Septicemia (except in labor)<br>Family history of emphysema
Lab test Variables	Number of abnormal lab test results<br>Number of normal lab test results<br>LDL-C cholesterol<br>Albumin lab: abnormal<br>Albumin/Creatinine lab: abnormal<br>Aspartate aminotransferase lab: normal<br>Basophils lab: normal<br>Bilirubin lab: abnormal<br>Bilirubin lab: normal<br>C reactive protein lab: abnormal<br>Calcium lab: normal<br>Eosinophils lab: abnormal<br>Eosinophils lab: normal<br>Fibrinogen lab: abnormal<br>Glomerular filtration rate lab: abnormal<br>Glucose lab: abnormal<br>Lymphocytes lab: abnormal<br>Monocytes lab: abnormal<br>Potassium lab: normal<br>Prostate specific Ag lab: normal<br>Protein lab: abnormal<br>Thyroxine lab: abnormal<br>Triglycerides: normal<br>Triglycerides: abnormal<br>Urea nitrogen lab: abnormal

Medication Variables	Total number of medications prescribed<br>GPI4: ACE Inhibitors<br>GPI4: Aminoglycosides<br>GPI4: Aminopenicillins<br>GPI4: Angiotensin II Receptor Antagonists<br>GPI4: Antidementia Agents<br>GPI4: Antidiarrheal Combinations<br>GPI4: Antihistamines - Non-Sedating<br>GPI4: Antihyperlipidemics - Combinations<br>GPI4: Antipsychotics - Misc.<br>GPI4: B-Complex with C<br>GPI4: B-Complex with Folic Acid<br>GPI4: Beta Blockers Cardio-Selective<br>GPI4: Beta-blockers - Ophthalmic<br>GPI4: Bulk Chemicals - C's<br>GPI4: Bulk Laxatives<br>GPI4: Central Muscle Relaxants<br>GPI4: Cephalosporins - 3rd Generation<br>GPI4: CMV Agents<br>GPI4: Cough/Cold/Allergy Combinations<br>GPI4: Emollient/Keratolytic Agents<br>GPI4: Emollients<br>GPI4: Erythromycins<br>GPI4: H-2 Antagonists<br>GPI4: HMG CoA Reductase Inhibitors<br>GPI4: Immunosuppressive Agents<br>GPI4: Infant Care Products<br>GPI4: Iron<br>GPI4: Leprostatics<br>GPI4: Loop Diuretics<br>GPI4: Nasal Anti-allergy<br>GPI4: Ophthalmic Immunomodulators<br>GPI4: Penicillin Combinations<br>GPI4: Salicylates<br>GPI4: Surfactant Laxatives<br>GPI4: Thiazides and Thiazide-Like Diuretics<br>GPI4: Vitamins with Lipotropics<br>GPI4: Water Soluble Vitamins

Abbreviations: Ag, antigen; ASCVD, atherosclerotic cardiovascular disease; BP – blood pressure; CCS, Clinical Classification Software; CMV – cytomegalovirus; GPI, Generic Product Identifier; HDL, high-density lipoprotein; HIV, human immunodeficiency virus; HMG CoA, 3-hydroxy-3-methyl-glutaryl-coenzyme A; LDL-C, low-density lipoprotein cholesterol; PCE, pooled cohort equations
